# Supplementary material for: Rapid Evolutionary Adaptation to Diet Composition in the Black Soldier Fly (Hermetia illucens)
Source: Insects. 2023 Oct 18;14(10):821. doi: 10.3390/insects14100821 (PMC10607891; doi:10.3390/insects14100821)
Supplement: Supplementary file 1 [file insects-14-00821-s001.zip › Table S1.pdf]

Table S1: Raw data from estimating juvenile's dry mater content during a pilot trial

Sample description	Tray weight (g)	Sample weight (g)	Weight after 105°C (Tray + sample, g)	Dry matter (%)
Juveniles	3.12	45.32	16.50	30%
Juveniles	3.13	61.06	21.07	29%
Juveniles	3.32	52.58	19.40	31%
Juveniles	3.13	53.69	19.32	30%
Juveniles	3.14	57.46	20.05	29%
Juveniles	3.13	43.15	16.18	30%
