# Supplementary figures and images for: Rapid Evolutionary Adaptation to Diet Composition in the Black Soldier Fly (Hermetia illucens)
Source: Insects. 2023 Oct 18;14(10):821. doi: 10.3390/insects14100821 (PMC10607891; doi:10.3390/insects14100821)

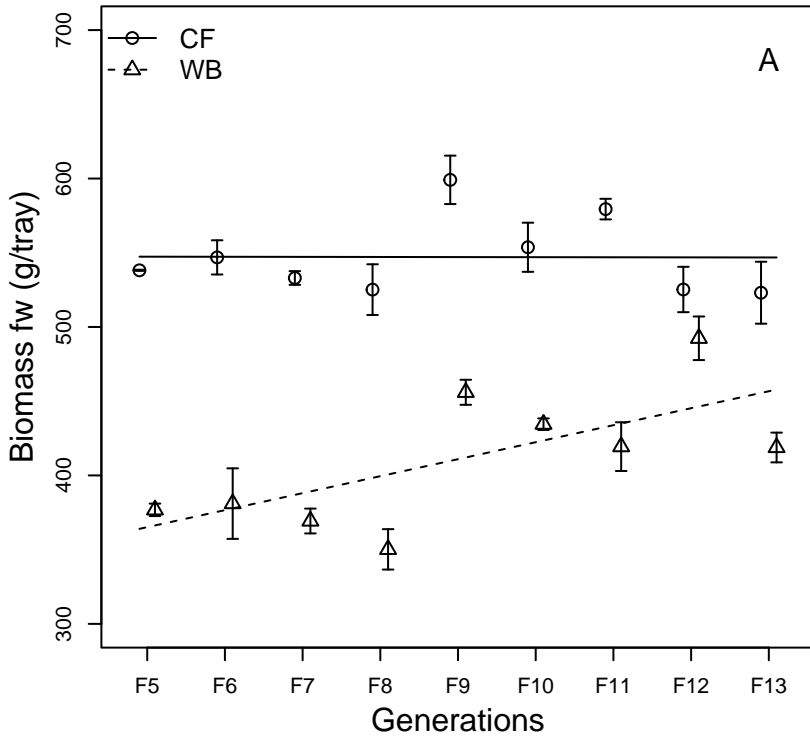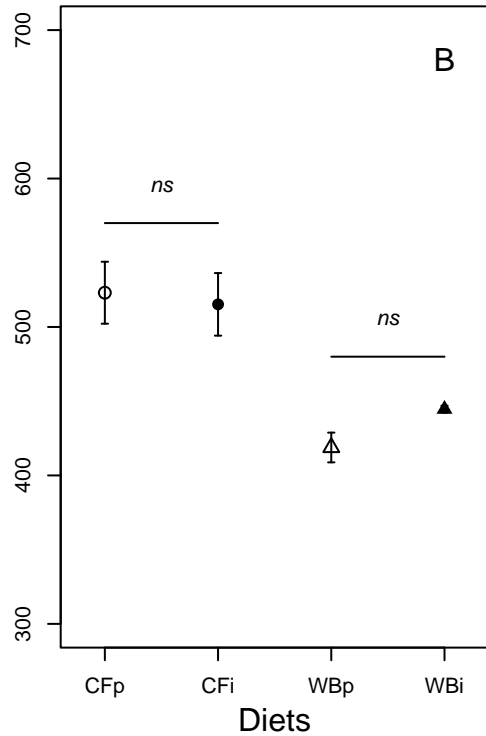

Supplement: Supplementary file 1 [file insects-14-00821-s001.zip › Figure S1.pdf]

Frass fw (g/tray)

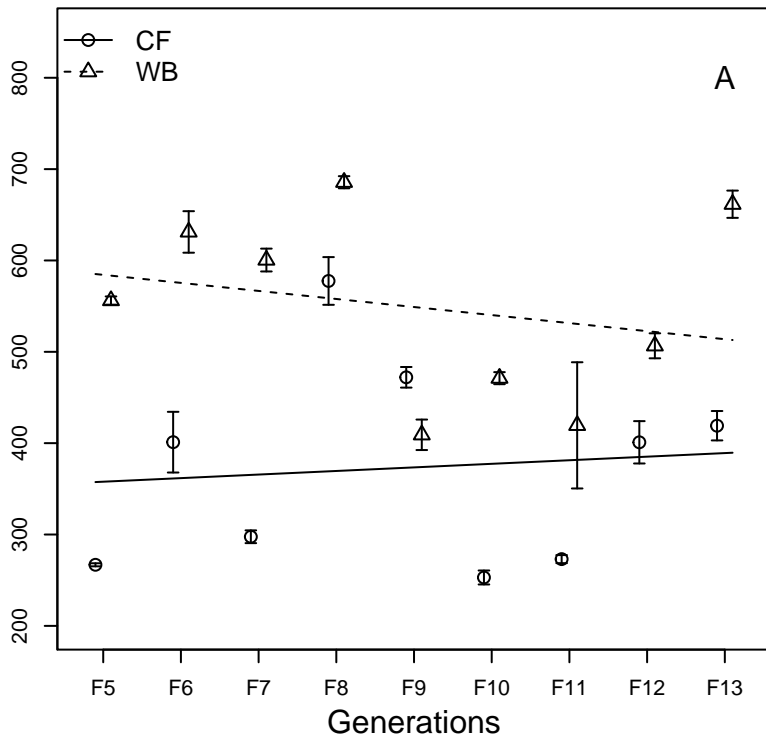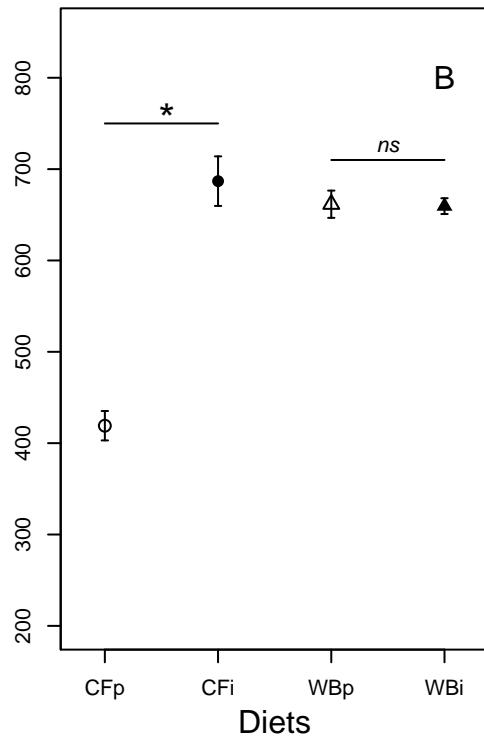

Supplement: Supplementary file 1 [file insects-14-00821-s001.zip › Figure S2.pdf]

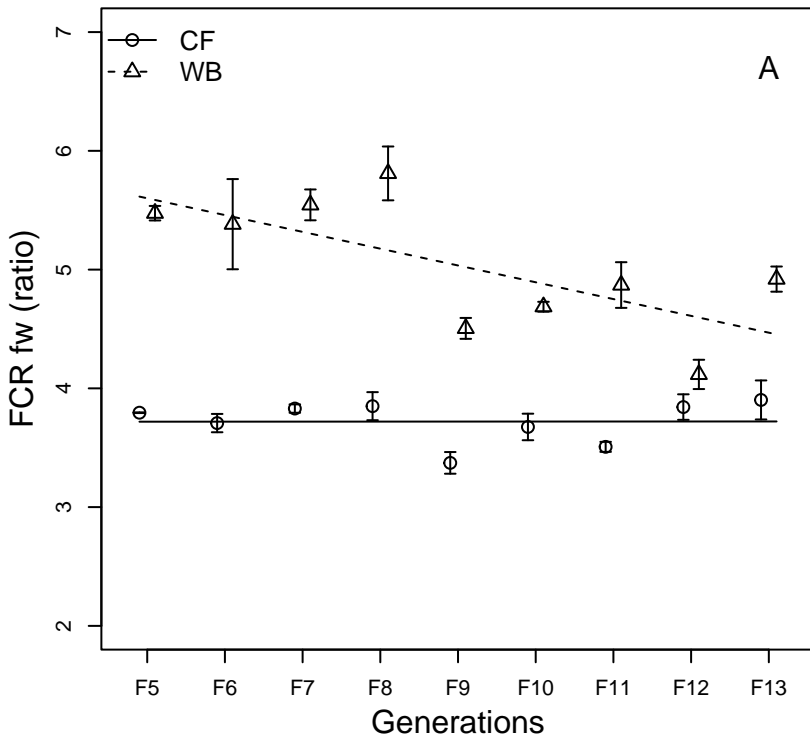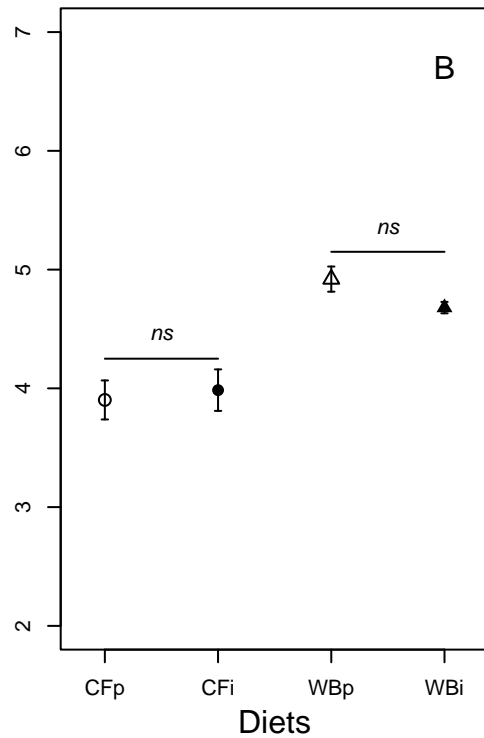

Supplement: Supplementary file 1 [file insects-14-00821-s001.zip › Figure S3.pdf]
